# A non-randomized, controlled, interventional study to investigate the effects of community pharmacists’ cognitive behavioral therapy–based interventions on medication adherence and relevant indicators in patients with depression

**DOI:** 10.1186/s12888-023-04602-5

**Published:** 2023-02-24

**Authors:** Masaki Shoji, Hatsuyo Maeda, Fumiyuki Watanabe, Kazunori Tanuma, Atsuko Fujiwara, Yusuke Iwanaga, Atsushi Shimada, Mitsuko Onda

**Affiliations:** 1 Faculty of Pharmacy, Department of Social and Administrative Pharmacy, Osaka Medical and Pharmaceutical University, 4-20-1, Nasahara, Takatsuki, Osaka 569-1094 Japan; 2 Study Group for Patient Counseling Using CBT-A, 7-7-1 Narashinodai, Funabashi, Chiba 274-8555 Japan; 3 School of Pharmacy, Nihon University, 7-7-1 Narashinodai, Funabashi, Chiba 274-8555 Japan; 4 Kamegaya Co. Ltd (Fit Care Depot), Shinyokohama TECH Bldg. A8F, Shinyokohama 3-9-18, Kohoku-ku, Yokohama, Kanagawa 222-0033 Japan; 5 Apis Pharmacy Co. Ltd., Nomura Hudousan Nishi Umeda Bldg, 2-1-22, Umeda, Kitaku, Osaka city, Osaka 530-0001, Japan

## Abstract

**Background:**

The prevalence of depression is increasing in Japan. Pharmacists play an important role in helping patients use medicines effectively. Several studies had investigated the impact of community pharmacists on patient adherence to antidepressant therapy, and their results indicated that further study was warranted.

**Methods:**

This study was conducted from June 2019 to May 2020 using a cluster non-randomized, open-label, parallel-group design. Four community pharmacy stores in Osaka and Hyogo Prefectures, Japan, participated in the study, and enrolled patients with unipolar depression. In the intervention group (IG), patients received cognitive behavioral therapy (CBT)-based medication support, and their medication adherence and adverse drug reactions were monitored by telephone. In the control group (CG), the pharmacists engaged in routine interactions with the study participants. Before participating in this study, the intervention-group pharmacists attended a 5-hour training session on CBT-based medication support. The primary outcome of this study was medication adherence, assessed using the Drug Attitude Inventory (DAI)-10. Secondary outcomes included the changes from baseline at 6 months in the following variables: the Patient Health Questionnaire (PHQ)-9 total score, the EQ-5D-5 L (Euro-QOL 5 dimensions 5 levels) score, patient satisfaction, and the Pharmacists’ Confidence Scale about Medication Consultation for Depressive Patients (PCMCD) score.

**Results:**

Four pharmacies (two in IG and two in CG) completed the intervention period. Results were obtained from 19 patients in the IG and 12 patients in the CG. In the IG, the mean DAI-10 score increased from 4.941 at baseline to 6.105, the mean PHQ-9 score decreased from 9.263 to 8.625, and the mean patient satisfaction score increased from 39.947 to 42.211. In the CG, the mean DAI-10 score decreased from 6.333 to 4.167, the mean PHQ-9 score increased from 9.333 to 12.923, and the mean patient satisfaction score decreased from 38.929 to 38.167.

**Conclusion:**

CBT-based medication support provided by community pharmacists may improve patient medication adherence to antidepressant therapy and symptoms. Such support can be expected to facilitate better treatment of depressed patients and may also allow the duration of treatment to be shortened.

**Trial registration:**

UMIN000037954, Date of first registration: 17/06/2019.

**Supplementary information:**

The online version contains supplementary material available at 10.1186/s12888-023-04602-5.

## Introduction

The World Health Organization estimated that 264 million people had depression worldwide in 2017, and the global burden of depression is on the rise [1]. In Japan, 1.276 million patients were diagnosed in 2017 with depression or other mood disorders, and the prevalence of depression has been increasing [2]. Depression greatly decreases the patients’ quality of life [3], and is a major cause of suicide. It also impairs job performance, leading to a significant loss in work productivity [4].

Depression generally require a long-term treatment. Treatment of major depression typically consists of three distinctive phases: acute, continuation, and maintenance [5]. The acute phase refers to the first 6 to 12 weeks of treatment until remission is reached. The continuation phase lasts for 4 to 9 months between remission and the diagnosis of recovery. The maintenance phase refers to the period after recovery is diagnosed. The use of antidepressants is recommended for the first 1 to 2 years of the maintenance phase. Relapse and recurrence prolong pharmacological treatment, and post-remission continuation of antidepressant treatment reduces their probability [6].

Patient adherence to the antidepressant regimen is a critical factor in effective management of symptoms. However, studies show that antidepressant regimens are not sufficiently complied with in many parts of the world. According to Masand, obstacles to adequate antidepressant therapy may involve issues that are attributable to physicians (e.g., inadequate patient education), medications (e.g., adverse drug reactions, delayed onset of action), and patients (poor motivation to continue therapy, concerns about treatment) [7]. Pharmacists are an important healthcare resource that helps patients understand the importance of medication and alleviate their concerns about drugs. The effects of pharmacist intervention on antidepressant adherence and clinical symptoms have been studied widely [8]. For example, Al-Saffar et al. have used pamphlet-based interventions [9]; and Brook et al. and Aljumah et al. have sought to improve medication adherence by coaching [10] and by using Shared Decision Making techniques [11], respectively. A few studies have been conducted in which pharmacists have modified the doses and prescriptions according to protocols, and provided supplementary prescriptions as needed [12–14]. However, there have been no studies to date that focus on patients’ biases in thinking and mood as factors contributing to poor adherence and try to solve these problems with CBT-based interventions, except for reports on RCT protocol [15].

Against this background, we hypothesize the following.

1. Taming their cognitive distortions would help patients adhere to their medication and treatment regimens and alleviate their illnesses.
2. Patient satisfaction with pharmacy services will increase as a result of the aforementioned supports.
3. Pharmacists’ confidence in dealing with depressed patients will increase as they gain experience with the interventions being introduced in this study.

## Objectives

The objective of this study was to investigate whether CBT-based medication support by the community pharmacist would improve the medication adherence, medical status, satisfaction towards the services of pharmacy of patients with unipolar depression. Another objective was to observe changes in pharmacists’ confidence in dealing with patients after gaining experience with the interventions introduced in the study.

## Methods

### Study design

This study was a cluster non-randomized, open-label, parallel-group, controlled study, based on the transparent reporting of evaluations with nonrandomized designs statement. This study was conducted from June 2019 to May 2020. Four community pharmacies in Osaka and Hyogo Prefectures, Japan, participated. This study was registered with the University hospital medical information network (UMIN) center (UMIN000037954, Date of first registration: 17/06/2019).

### Participants

#### Pharmacists inclusion criteria

The inclusion criteria for pharmacists included (i) employed at a community pharmacy that accepts prescriptions mainly from psychosomatic medicine and psychiatry, (ii) routinely provides medication to depressive patients, and (iii) has received an explanation of the purpose of the research from the Investigator, understands the explanation, and wishes to participate of their own will. The type and length of the pharmacists’ work experiences were not taken into account. No particular exclusion criteria were defined. Volunteering pharmacists were explained about the objectives and other details of this study verbally and through written documents, and signed the informed consent form before participating in this study. Before the start of the study interventions, the study investigators met the psychosomatic medicine specialists whose prescriptions were processed by the participating pharmacies, and explained the objectives and other details of this study to obtain their consent to assist with this study.

#### Patients inclusion criteria

Patients were eligible for inclusion if they were (i) diagnosed with unipolar depression, (ii) currently receiving antidepressant prescriptions, and (iii) visiting the participating pharmacy for prescription refills. As part of the patient recruitment process, the participating pharmacist contacted the prescribing physician, and checked the diagnosis of the consenting patient.

#### Patients exclusion criteria

Exclusion criteria included (i) patients suspected of having maniac episodes, (ii) diagnosis of schizophrenia, (iii) diagnosis of dementia, and (iv) patients not visiting the pharmacy in person. Patients were eligible if their pharmacy visit was accompanied by a helper.

### Allocation

This was a non-randomized study. One of the study investigators (AF) allocated the participating pharmacies to the intervention and control groups. For this purpose, the prescribing practitioners’ receptiveness for the pharmacist interventions, the personal relationship between the pharmacist and patients, and similarity in pharmacy size between the two groups were taken into consideration.

#### Intervention group

In the intervention group, patients received CBT-based medication support, and their medication adherence and adverse drug reactions were monitored by telephone calls.

#### Control group

Control-group pharmacists used their routine interactions with their patients.

### Educational program

Before participating in this study, pharmacists attended a 5-hour training course on CBT-based medication support. This training program was developed by Tanuma et al. with a focus on community pharmacists [16, 17]. It was informed by the “Kokoro-no-skill-up Training” (emotional and communication skills training) module developed by the National Center for Cognitive Behavior Therapy and Research, one of the institutes of the National Center for Neurology and Psychiatry. Specifically, this program aimed to support patients (i) identify the association between their thought patterns and moods, (ii) find objective counter-evidence that refutes their negative thought patterns, (iii) replace unrealistic and non-adaptive thinking styles with realistic and adaptive ones, and (iv) achieve a better mood. This program included many roll-play sessions to help attendees actively participate and improve practical skills. The original program takes 8 h, but in this study, a 5-hour version was implemented. The 5-hour program differs from the original in the following;

1. The commentary on building basic communication with patients was shortened.
2. The role-playing cases were limited to depressed patients.
3. Added detailed commentary on how to deal with depressed patients.

The role-plays were conducted in pairs of three, with the patient, pharmacist, and observer roles, respectively. The person playing the role of the patient responded to the person playing the role of the pharmacist according to his or her preferences. The concept is that the pharmacist uses cognitive reconstruction skills to elicit evidence from the patient’s thoughts and moods, guiding the patient to recognize his or her own rebuttal to the evidence. Observers provide feedback after the role-play. After the role-play, each of the three participants discussed their impressions, what the person who played the role of the pharmacist aimed to achieve, and areas for improvement. The program also uses an original thought record table, and the pharmacists learned how to use it. An example of a role-play and a thought record table are provided in Supplemental Figs. 1 and 2.

### Outcomes

#### Primary outcome

The primary outcomes of this study were medication adherence. Medication adherence was evaluated using the Drug Attitude Inventory (DAI)-10 questionnaire (Japanese version).

The DAI-10 questionnaire is a 10-item self-report scale widely used to evaluate patients’ attitudes towards medication. Respondents answer each question by choosing true or false. Each item is scored 1 or − 1, with the total score ranging from − 10 (very poor attitude) to + 10 (best possible attitude). A positive total score indicates a positive subjective response (adherent), whereas a negative total score indicates a negative subjective response (non-adherent) [18]. The Japanese version of this questionnaire has been validated for internal consistency and test-retest reliability [19]. The DAI is widely used as a measure of medication adherence for a variety of psychiatric and neurological disorders, including depression [20], substance abuse disorders, schizophrenia, bipolar disorders, personality disorders, and anxiety disorders [21]. The DAI-10 was selected as the primary outcome measure because of its lower burden on patient responses compared to other assessment instruments with a larger number of questions.

#### Secondary outcomes

The secondary outcomes of this study were assessed using the Patient Health Questionnaire (PHQ)-9, patient satisfaction [22], the EQ-5D-5 L (Euro QOL 5 dimensions 5 levels), and the Pharmacists’ Confidence Scale About Medication Consultation for Depressive Patients (PCMCD) scales. The PHQ-9 scale [23] was used to evaluate patient conditions. In this study, its validated Japanese version was used [24]. The PHQ-9 scale was recommended for evaluating antidepressant treatment effects in the guidelines published by the National Institute for Health and Clinical Excellence, UK [25]. The smaller scores indicate better conditions. The EQ-5D-5 L scale (Japanese version) was used to evaluate the patient quality of life [26]. A questionnaire consisting of a 10-item, 5-point Likert scale developed by Pedro et al. was used to assess patient satisfaction. The score is expressed as the mean of the 10 items, with a maximum score of 5.0 and a minimum score of 1.0. Higher values indicate higher satisfaction [22]. The PCMCD scale developed by Shoji et al. was used to evaluate pharmacists’ self-reported competence in medication consultation [27] The PCMCD questionnaire consists of 12 questions, and has the following subscales: relationship building, comprehension of condition, and information provision. The higher scores indicate greater confidence.

### Statistical analysis

The differences in the study variables between the start (baseline) and end of the study (6-months post-baseline) were evaluated in the per-protocol set. The Between-group differences in the 6-month changes in DAI-10, PHQ-9, and EQ-5D-5 L and PCMCD scores were compared using the unpaired t-test. To examine the factors that influence the DAI-10 scores and their strengthes, a multiple regression analysis was performed with the change in the DAI-10 as the dependent variable.

All statistical analyses were performed using the IBM SPSS Statistics Version 26 (IBM Japan, Tokyo).

## Risk management

We had cognited following latent risks in this study;

1. worsening the relationship between the patients-pharmacist or physician-pharmacist due to the failure or wrong way of CBT-based medication support.
2. 1 might worsen the patient’s symptoms.

We explained these to the pharmacist in advance, and instructed to exclude the patient from intervention if these happened.

## Results

### Study flow

**Fig. 1:**
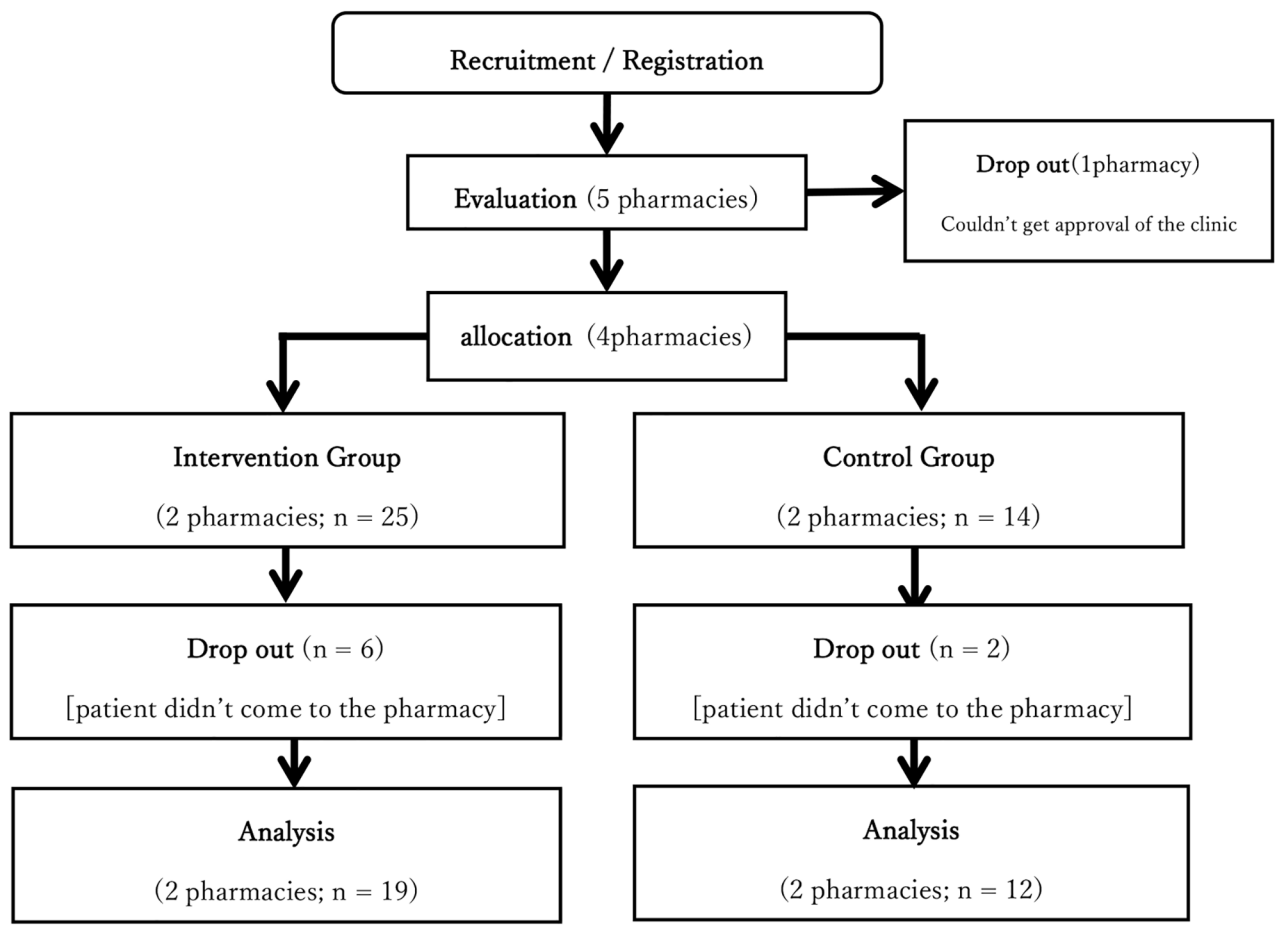
Flow of the study

Five pharmacies participated in this study. However, one of them withdrew before the start of the study because the prescribing practitioners did not consent to provide assistance to this study. The remaining four pharmacies were allocated to the intervention and control groups at a 1-to-1 ratio. During the study, 6 patients in the intervention group and 2 in the control group dropped out. Results were obtained from 19 intervention-group and 12 control-group patients (Fig. 1).

### Patient attributes

Table 1 summarizes the background and demographic attributes of the study patients. The age, sex, number of years of illness, presence/absence of comorbidity (yes or no), and employment status (employed or unemployed) were evenly distributed between groups. During the intervention period, the mean (SD) number of visits to the pharmacy was 10.0 (5.8) for the intervention group and 11.1 (4.4) for the control group (p = 0.58).

**Table 1:** Patient attributes

Patient attributes	IG	CG	p
Age [Mean(SD)]	48.5 (14.5)	49.4 (11.7)	0.861
Sex [Female(%)]	57.9	50.0	0.653
Number of years affected [ Mean (SD)]	3.9 (4.8)	6.4 (3.7)	0.168
Presence of comorbidity (%)	63.2	50.0	0.450
Occupation (%)	63.6	30.8	0.086

IG: Intervention group, CG: Controlled group,

### Changes in outcomes in patients

Table 2 shows the mean primary and secondary outcome scores by time point and group. In the intervention group, the mean DAI-10 score increased from 4.941 at baseline to 6.105, the mean PHQ-9 score decreased from 9.263 to 8.625, and the mean patient satisfaction score increased from 3.995 to 4.221. In the control group, the mean DAI-10 score decreased from 6.333 to 4.167, the mean PHQ-9 score increased from 9.333 to 12.923, and the mean patient satisfaction score decreased from 3.893 to 3.817. Neither group showed a noteworthy change during the study in the mean EQ-5D-5 L score.

**Table 2:** Changes in Outcome scores

	Baseline		After 6 months		Difference^#^		**P**
	IG	CG	IG	CG	IG	CG	
DAI-10	4.941	6.333	6.105	4.167	1.059	-2.000	0.035
PHQ-9	9.263	9.333	8.625	12.923	-0.813	2.818	0.049
EQ-5D-5 L	0.764	0.630	0.857	0.733	0.093	0.129	0.748
Satisfaction	3.995	3.893	4.221	3.817	0.226	-0.142	0.127

#: Difference between 6months and baseline, DAI-10: Drug Attitude Inventory-10, PHR-9:

Patient Health Questionnaire-9, EQ-5D-5 L: Euro Qual 5 Dimensions 5 Level

### Effect of each variable on the amount of change in the DAI-10

A multiple regression analysis was performed using a stepwise method with the change in DAI-10 as the dependent variable and PHQ-9, EQ-5D, patient satisfaction, as independent variables. As a result, only the change in patient satisfaction (standardized coefficient β: 0.450, p = 0.024) was included in the model. The adjusted R-squared value was 0.203. A list of the excluded variables and their variable correlation coefficients and significance probabilities is provided as a supplemental document.

### Pharmacist attributes

Five pharmacists in IG and three pharmacists in CG provided patient care in this study and responded to the questionnaire for pharmacists. 60.0% of the IG and 33.3% of the CG were female. The mean years of experience as a pharmacist was 10.1 for the intervention group and 3.3 for the control group (p = 0.20).

### Changes in outcomes in pharmacists

Post-baseline changes in the participating pharmacists’ self-reported confidence in interacting with patients with depression were analyzed, and the results are summarized in Table 3. The intervention group showed increases in all the three subscale scores, with a marked increase in the relationship building. By contrast, the control group showed a mild decrease in the relationship building subscale, but no changes were observed in others.

**Table 3:** Changes in Study Pharmacists’ Self-Reported Confidence in Interacting With Patients With Depression (PCMCD Score)

	Baseline		After 6 months		Difference^#^		**P**
	IG	CG	IG	CG	IG	CG	
Comprehension of condition	9.0	10.7	10.8	10.7	2.0	0.0	0.145
Information provision	10.6	11.0	12.6	11.0	1.8	0.0	0.142
Relationship building	6.0	6.3	7.2	6.0	1.2	-0.3	0.041

#: Difference between 6months and baseline

## Discussion

This study suggested the possibility that community pharmacists can help improve the medication adherence of patients with unipolar depression by providing CBT-based medication support. This possibility is probably associated with intervention-induced changes in the patients’ perceptions of medication and health care professionals. This parallel-group study to evaluate the impact of CBT-based medication support on antidepressant adherence was the first of its kind in Japan. The intervention-group patients of our study showed improvements in clinical symptoms, and this positive finding contradicted the results of the systematic meta-analysis that reviewed the effects of pharmacist intervention on antidepressant adherence and symptomatology [8]. We speculated that this change may due to patients learning how to self-management with CBT through the interventions.

Given that CBT-based counseling support can improve medication adherence and symptoms, this approach will provide better healthcare opportunities than information-focused patient education. One of the major challenges for CBT-based medication support is to elicit unspoken (and often biased) assumptions and thoughts from the patients during the contact. Building trust, therefore, is the foundation for the community pharmacist to provide CBT-based medication support to their patients. The intervention-group patients also exhibited increased patient satisfaction. Moreover, the intervention-group pharmacists demonstrated an increase in the self-confidence in interacting with patients with depression. Intervention-group pharmacists commented that their patient assignment helped them build trusting relationships because it allowed them to have a stronger commitment to their study patients. Specifically, the study setting enabled them to gather information on their patients’ background, medical history, and other personal matters. Effective interventions based on these pieces of information contributed to the patients’ satisfaction and the pharmacists’ sense of competence.

Several methodological limitations of this study must be pointed out. First, this was not a randomized trial, although the distributions of the demographic and baseline characteristics of the study patients were not significantly biased. The proportion of patients with employment was slightly higher in the intervention group. This probably reflected the differences in the geographic location, with the intervention-group pharmacies being placed in a more urban area than their counterparts. Second, the sample size of this study was small. This warranted a larger study in the future. Third, the primary and secondary endpoints were subjective. However, the instruments used in this study have been thoroughly tested for validity and reliability, and are commonly used in research. Fourth, patient selection bias may have occurred. Fifth, this study included both newly and previously diagnosed patients. As a result, the study patients had varying lengths of disease duration. However, our multiple regression analysis showed a poor correlation between the post-baseline DAI-10 score change and disease duration (Supplemental Table 1), and these results suggested the positive effects of the CBT-based medication support were independent of the time from diagnosis. Patients with depression tend to have negatively biased thinking styles regardless of the duration of disease. CBT can help patients learn effective self-help skills, thereby contributing to preventing the recurrence of depression. Sixth, some control group pharmacists attended the training. The control group was asked not to use CBT-A during the intervention period and to treat patients as usual, but it cannot be denied that this may have been weakened the intervention effect of this study. This study did not collect data on the relapse or recurrence of depressive symptoms. Seventh, the intervention group had telephone follow-up as needed, which may have increased patient contact. It is undeniable that this may have affected medication adherence and patient satisfaction. However, telephone follow-up of patients in intervention trials has been conducted in previous studies [12–14] and is not unique to this study. A longer, randomized study in the future will help clarify the potential clinical effects of CBT-based medication support.

Despite the above limitations, this interventional study demonstrated that CBT-based interventions by community pharmacists improved the medication adherence of patients with depression. This also suggests that it might be possible to shorten the duration of treatment for depressed patients and thus reduce health care costs. We hope that similar initiatives will be pursued in pharmacies in the future for patients with panic disorder, insomnia, addiction, and other disorders.

We believe that it would be beneficial to provide basic education on CBT to pharmacy students at universities so that medication support based on CBT can be practiced more widely in the community. It would be also desirable to establish an organization, led by a pharmacist association, to support pharmacists in clinical settings to continue learning about CBT and to monitor the adequacy of the support provided.

## Electronic supplementary material

Below is the link to the electronic supplementary material.

Supplementary Material 1 An example of a role play conducted during the training

## Data Availability

The datasets used and/or analysed during the current study available from the corresponding author on reasonable request.
