# Supplementary material for: A non-randomized, controlled, interventional study to investigate the effects of community pharmacists’ cognitive behavioral therapy–based interventions on medication adherence and relevant indicators in patients with depression
Source: BMC Psychiatry. 2023 Feb 24;23:124. doi: 10.1186/s12888-023-04602-5 (PMC9951417; doi:10.1186/s12888-023-04602-5)
Supplement: Supplementary file 1 — Supplementary Material 1 An example of a role play conducted during the training [file 12888_2023_4602_MOESM1_ESM.docx]

**Supplemental Figure 1. An example of a role play conducted during the training**

In the training for the pharmacists, the following dialog was presented as a model example of CBT-based medication support.

Pharmacist: Mr. Noguchi, you seem a little pale. Are you taking your medication?

Patient: That's...I'm a bad person. I can't even take my medication properly.

Pharmacist: At all? What makes you think that?

Patient: Yesterday I went out to lunch and forgot to bring my medication with me.

Pharmacist: Do you always forget your lunchtime medication?

Patient: No, it was the only time I forgot. But I was told by the doctor and the pharmacist, and I was trying to be careful, so I feel ashamed that I forgot....

Pharmacist: Oh, I see, you were careful and forgot to bring your medications and felt miserable?

Patient: Yes, that's right.

Pharmacist: What do you do on a daily basis to try to make sure you don't forget to take your medication?

Patient: Well, I use my medication calendar and try to make sure I take medicine.

Pharmacist: I understand it must be disappointing if you forgot to take your medication, even though you are trying so hard every day to stick to the schedule. By the way, you have been taking this medicine for about 3 months now. How many times have you forgotten to take it?

Patients: Um, it is still just this one time.

Pharmacist: Well...does the fact that I couldn't do it just once in 3 months mean that I can't do it at all?

Patient: Well, no, it is not impossible at all.

Pharmacist: Yes - so are you a bad person?

Patient: No... I was feeling sorry for myself, depressed, and I was starting to feel like a very bad person. But I remembered that I have been trying not to forget to take my medication, and I also remembered that I am usually taking it properly, so that gives me confidence. The medication seems to be working, and I will continue trying my best to take in on schedule. Thank you.

**Supplemental Figure 2. Column table created from role-play scenarios**


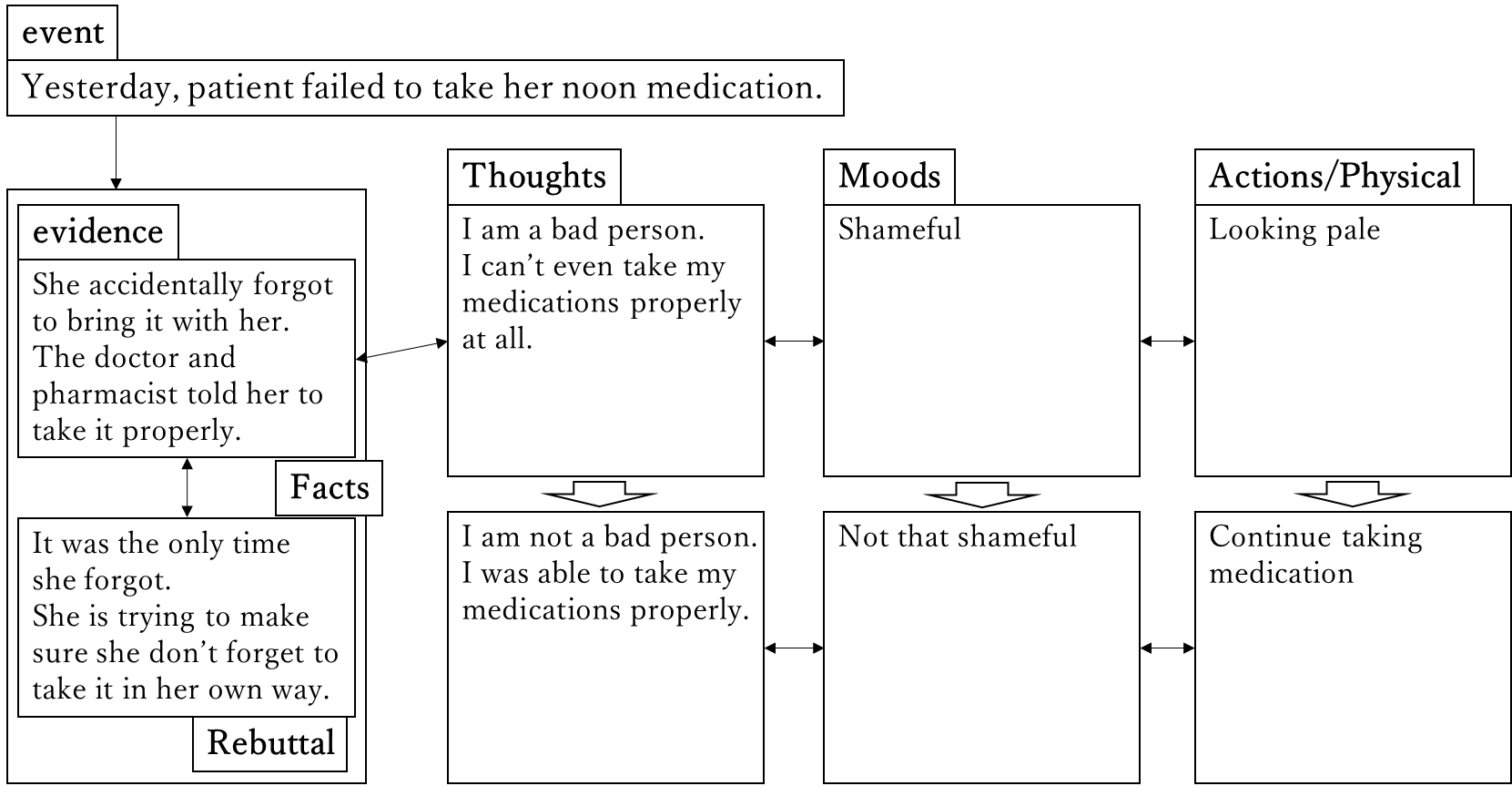


**Supplemental Table 1**

| **Excluded variables ^a^** | | | | | |
| --- | --- | --- | --- | --- | --- |
| model | Standard regression coefficients for the input | t value | p | Partial correlation | Statistics of collinearity |
|  |  |  |  |  | Tolerance level |
| ∆PHQ-9 | -.281 | -1.495 | .149 | .304 | .934 |
| ∆QOL | .046 | .233 | .818 | .050 | .933 |
| a. dependent variable: ∆DAI－10 score | | | | | |
